# Quantitative trait locus analysis of heterosis for plant height and ear height in an elite maize hybrid zhengdan 958 by design III

**DOI:** 10.1186/s12863-017-0503-9

**Published:** 2017-04-17

**Authors:** Hongjian Li, Qingsong Yang, Nannan Fan, Ming Zhang, Huijie Zhai, Zhongfu Ni, Yirong Zhang

**Affiliations:** 1 State Key Laboratory for Agrobiotechnology and Key Laboratory of Crop Heterosis Utilization (MOE), China Agricultural University, Beijing, 100193 China; 2 National Maize Improvement Center of China, China Agricultural University, Beijing, 100193 China

**Keywords:** Heterosis, Plant height, Ear height, Design III, Maize

## Abstract

**Background:**

Plant height (PH) and ear height (EH) are two important agronomic traits in maize selection breeding. F_1_ hybrid exhibit significant heterosis for PH and EH as compared to their parental inbred lines. To understand the genetic basis of heterosis controlling PH and EH, we conducted quantitative trait locus (QTL) analysis using a recombinant inbreed line (RIL) based design III population derived from the elite maize hybrid Zhengdan 958 in five environments.

**Results:**

A total of 14 environmentally stable QTLs were identified, and the number of QTLs for Z_1_ and Z_2_ populations was six and eight, respectively. Notably, all the eight environmentally stable QTLs for Z_2_ were characterized by overdominance effect (OD), suggesting that overdominant QTLs were the most important contributors to heterosis for PH and EH. Furthermore, 14 environmentally stable QTLs were anchored on six genomic regions, among which four are trait-specific QTLs, suggesting that the genetic basis for PH and EH is partially different. Additionally, *qPH.A-1.3*, modifying about 10 centimeters of PH, was further validated in backcross populations.

**Conclusions:**

The genetic basis for PH and EH is partially different, and overdominant QTLs are important factors for heterosis of PH and EH. A major QTL *qPH.A-1.3* may be a desired target for genetic improvement of maize plant height.

**Electronic supplementary material:**

The online version of this article (doi:10.1186/s12863-017-0503-9) contains supplementary material, which is available to authorized users.

## Background

Maize is one of the most important crops worldwide, which serves as food, animal feed and raw materials of bioenergy. Plant height (PH) and ear height (EH) are two main selection factors in maize architecture because optimal PH and EH are critical for improving plant density to maximize the utilization of fertilizer, moisture and incident photosynthetically active radiation [1–3]. More than 40 maize dwarf genes for PH have been cloned in maize so far, which were reported to be related to various biosynthesis pathways [4–12]. However, these mutants have less potential applications in maize breeding because of their harmful impacts on grain yield [13]. An alternative strategy is to identify moderate alleles (QTLs) reducing plant height, which may be feasible and effective. Since the first publication of molecular linkage of maize, a number of QTLs for plant height and ear height have been reported [14–17]. Wang et al. integrated QTLs for plant height and ear height based on the target map IBM2 2008 Neighbors. They found several GA pathway genes were located in the meta-QTL region [18]. Xing et al. cloned a major plant height QTL-*qph1*, which contains a naturally occurring rare SNP in *br2. qph1* reduced plant height and ear height with no or very little negative impact on yield when heterozygous [19]. *ZmGA3ox2*, which is a candidate gene for a major QTL-*qPH3.1*, was also reported to modify approximately 10% of the total plant height without influence on grain yield, yield-associated traits or flowering time [20]. The identification of more QTLs/genes related to the two traits might facilitate our understanding of the genetic mechanism of height development and benefit future maize improvement.

The superior performance of F_1_ hybrid over its parental lines was defined as heterosis, which has been led to great success in plant breeding [13, 21]. Maize PH and EH exhibit significant heterosis and can be easily and accurately measured [22–25]. Thus, QTL mapping of heterotic loci for the two traits has attracted much attention. Up to date, design III and triple testcross design (TTC) are most commonly used experimental designs for estimating the average degree of dominance or overdominance of quantitative genes. Design III populations were constructed using F_n_ plants from a cross between two inbreds to back-crossed with the inbred parents, while TTC populations were constructed using F_n_ plants from a cross between two inbreds to back-crossed with the inbred parents and the hybrid [23, 26]. Stuber et al. did pioneering work in maize to identify QTLs related to heterosis with the aid of molecular markers. They concluded that overdominance (or pseudo-overdominance) was the main cause of heterosis for PH and EH [27]. Nevertheless, Cockerham and Zeng showed that dominance of favorable alleles together with epistatic between linked QTLs played important roles in the phenomenon of heterosis by reanalyzing Stuber’s data using design III [28]. Using a random-mated maize population, Lu et al. concluded that most of the QTLs for PH showed partial to complete dominance [25]. Frascaroli et al. studied heterosis underlying PH with the utilization of a triple testcross design (TTC) population and demonstrated that heterosis in the maize hybrid B73 × H99 was mainly due to dominance at various levels, with epistasis playing a less important role [23]. Also, Song et al. emphasized the predominance of overdominant QTLs for PH and EH, and they found three important heterotic regions for the two traits [22]. Although such studies have been reported, the ever changing conclusions of genetic basis underlying heterosis for maize PH and EH suggested that more investigations should be conducted.

The maize hybrid Zhengdan 958 is one of the most popular hybrids in China, which contributed about 20% of total maize production [29]. However, studies on heterosis for PH and EH of this hybrid were rarely reported. In the present study, we used a design III population from the hybrid Zhengdan 958 to analyze QTLs associate with heterosis. Our objectives were: 1) to assess the level of heterosis for PH and EH; 2) to detect the QTLs and evaluate their effects related to heterosis; 3) to validate QTL-*qPH.A-1.3* in backcross populations.

## Method

### Plant materials and field experiments

**Fig. 1:**
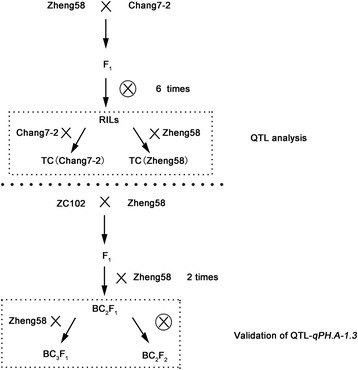
Experimental flow chat for QTL analysis and validation. The crossed of 162 RILs to their parental lines Zheng 58 (TC Zheng 58) and Chang 7–2 (TC Chang 7–2) were phenotyped for further QTL analysis. A major QTL, *qPH.A-1.3* was validated using BC_2_F_1,_ BC_3_F_1_ and BC_2_F_2_ populations

A total of 162 RILs derived from the hybrid Zhengdan 958 (Zheng 58 × Chang 7–2), were crossed with its two parents following the design III [23, 26]. In brief, 162 RILs (F_7_) derived from the hybrid Zhengdan 958 were used as pollen parents to cross the parental lines Zheng 58 [TC (Zheng 58)] and Chang 7–2 [TC (Chang 7–2)] (Fig. 1). The two populations of TC progeny along with other materials (i.e., the parental lines, Zhengdan 958 and RILs) were field-tested in five environments in China with three replications per location. Location-year information and climate data across the whole life cycle are presented in Additional file 1. Field management policies followed local standard practices.

The RIL line ZC102, which was homologous with the parental line Chang7-2 at the QTL-*qPH.A-1.3* region and shared 74% of the same genetic background with parental line Zheng 58, was chosen as the donor line to cross with Zheng 58 with marker assisted selection. In 2014, 350 BC_2_F_1_ plants were genotyped and field tested in Jilin province. Several heterozygous plants were self-pollinated or back crossed with Zheng 58 to produce BC_2_F_2_ and BC_3_F_1_ populations. In the winter of 2014, 217 BC_2_F_2_ and 161 BC_3_F_1_ individuals were planted in Hainan.

PH was scored as the distance from the soil line of the plant to the top of the tassel, and EH was measured as the distance from the soil to the primary ear node.

### Data analysis

Mid-parent heterosis (MPH) was used to score the percentage of heterosis: MPH = (F_1_-MP)/MP × 100, where MP represented the mid-parent value. Following the methods reported by Comstock et al. [26] and Melchinger et al. [30], the crosses of RILs to their parental lines Zheng 58 (TC Zheng 58) and Chang 7–2 (TC Chang 7–2) were denoted as L_1i_ and L_2i_ (i = 1 ~ 162), respectively. The linear transformations were Z_1i_ = (L_1i_ + L_2i_)/2 and Z_2i_ = L_2i_-L_1i_. A combined ANOVA over five environments was calculated to estimate variance components. Additive Variances (*V*_*A*_) within Z_1_ and dominance variances (*V*_*D*_) within Z_2_ were used to score the average degree of dominance D* as (*V*_*D*_/*2V*_*A*_) ^0.5^, which stood for the degree of dominance over all separating loci [23, 28, 30].

The best linear unbiased prediction (BLUP) values across five environments were computed with the PROC MIXED procedure in SAS (SAS Institute Inc., North Carolina, USA). Broad-sense heritability (*h*_*B*_^*2*^) were estimated as *h*_*B*_^*2*^ = *σ*_*g*_^*2*^/(*σ*_*g*_^*2*^ *+ σ*_*ge*_^*2*^ */n + σ* ^*2*^ */nr*), where *σ*_*g*_^*2*^ is the genetic variance, *σ*_*ge*_^*2*^ is the genotype by environment interaction variance, *σ* ^*2*^ is the error variance, *n* is the number of environments, and *r* is the number of replications of each experiment [31, 32]. Correlation coefficients among PH and EH were estimated using adjusted mean values for Z_1_ and Z_2_.

### Genotyping and linkage analyses

The RIL population was genotyped using a Maize SNP50 BeadChip [33]. A genetic linkage map was constructed using MSTMap software [34].In brief, a total of 905 SNP markers were mapped in the genetic linkage map with an average of 2.65 cM between adjacent markers, spanning 2402.0 cM (Additional file 7).

### QTL Analysis

For each Z_s_ (s = 1, 2) population, the mean of three replications in a single location were used for QTL analysis. The BLUP values across five environments were used for combined analysis. QTL analysis was performed through the composite interval mapping (CIM) using Windows QTL Cartographer version 2.5 [35, 36]. A test of 1,000 permutations was adopted to determine the thresholds for the logarithm of odds (LOD) scores of putative QTLs [32]. QTLs in Z_1_ and Z_2_ reflect the augmented additive effects *a*_*i*_ * and augmented dominance effects *d*_*i*_ *, respectively [30]. The dominance degree ratios were estimated as |*d*_*i*_ */*a*_*i*_ *| = augmented dominance effects/augmented additive effects: A, additive (|*d*_*i*_ */*a*_*i*_ *| ≤ 0.20); PD, partial dominance (0.20 < | *d*_*i*_ */*a*_*i*_ *| < 0.80); D, dominance (0.80 ≤ |*d*_*i*_ */*a*_*i*_ *| < 1.20); and OD, overdominance (|*d*_*i*_ */*a*_*i*_ *| ≥ 1.20). QTL were congruent with overlapping confidence intervals across environments for a given trait.

### SSR maker development

The stable QTL-*qPH.A-1.3* was identified between SNP markers *SNP5629* and *SNP6190*. Sequence information in this region was obtained from the maize sequence database (http://www.maizesequence.org/) to develop new markers. The sequences were scanned using the software SSRHunter1.3 [37] to detecte simple-sequence repeats (SSRs). Primers were designed by PRIMER 5.0 or PRIMER 3 (http://frodo.wi.mit.edu/primer3/) [38]. SSR primers appeared polymorphic between two parental lines were used for marker associated selection and genotyping of each plant in the BC_2_F_1_, BC_3_F_1_ and BC_2_F_2_ populations.

## Results

### Heterosis and population performance

The average field performance and heterosis of PH and EH for the basic populations are listed in Table 1. Chang 7–2 had higher PH and EH than Zheng 58 in all the five environments (*P* < 0.01). Compared to parental lines, the hybrid Zhengdan 958 showed overwhelming superiority in each environment, with heterosis ranged from 25.27% to 40.32% for PH and from 25.7% to 43.94% for EH.

**Table 1:** Performance of the basic generations (the parental line Zheng 58, Chang 7–2 and the hybrid Zhengdan 958) and heterosis for plant height (PH) and ear height (EH) in five environments

Trait	Environment	Zheng58	Chang7-2	MP	F_1_	MPH (%)
PH	E1	183.19 ± 10.03	199.86 ± 8.47^**,a^	191.52	268.75 ± 12.61^**,b^	40.32^c^
	E2	148.28 ± 11.40	175.32 ± 11.93^**^	161.80	209.67 ± 9.84^**^	29.59
	E3	175.37 ± 7.88	205.21 ± 11.97^**^	190.29	247.20 ± 12.77^**^	29.91
	E4	161.19 ± 7.63	210.50 ± 6.27^**^	185.84	247.97 ± 8.57^**^	33.43
	E5	180.52 ± 8.36	215.01 ± 6.88^**^	197.77	247.75 ± 6.96^**^	25.27
EH	E1	68.90 ± 7.95	102.81 ± 10.25^**^	85.86	123.58 ± 10.04^**^	43.94
	E2	45.63 ± 3.86	84.03 ± 6.09^**^	64.83	86.73 ± 7.72^**^	33.78
	E3	61.14 ± 6.65	101.26 ± 9.40^**^	81.20	114.26 ± 8.28^**^	40.71
	E4	51.07 ± 5.65	101.13 ± 2.86^**^	76.10	105.56 ± 4.84^**^	38.71
	E5	61.41 ± 5.57	104.43 ± 6.43^**^	82.92	104.23 ± 5.98^**^	25.70

^**^ *P* ≤ 0.01

^a^Comparison between Zheng 58 and Chang 7–2 using *t* test; ^b^Comparison between midparent (MP) and F_1_ using *t* test, ^c^Mid-parent heterosis (MPH): (F_1_-MP)/MP × 100

The minimum, maximum, mean values of TC populations for each trait are shown in Table 2. With respect to TC progenies, the average performance of TC (Chang 7–2) were significantly higher than TC (Zheng 58) for both PH and EH (*P* < 0.01), which is consistent with the observation of parental lines Chang 7–2 and Zheng 58. Correlation coefficients among PH and EH within TC populations were also tested. Interestingly, PH was positively correlated with EH in each TC population. Notably, drought stress could significantly decrease maize plant height [39]. The total precipitation in May of 2012 was extremely lower and no irrigation is given in time in E2, which may lead to the lowest plant height in F_1_ and TC populations.

**Table 2:** Performance of plant height (PH) and ear height (EH) for TC (Zheng 58) and TC (Chang 7–2) in five environments

Population	Phenotypic data	Environment				
		E1	E2	E3	E4	E5
				PH		
TC(Zheng 58)	Mean(cm)	238.80	187.61	226.48	221.29	225.55
	Max(cm)	275.48	235.24	270.47	259.83	264.5
	Min(cm)	196.43	158.47	178.4	187.79	185.78
TC(Chang 7–2)	Mean(cm)	248.75**^a^	210.02**	243.81**	243.32**	244.12**
	Max(cm)	297.38	242.29	279.2	283.13	274.67
	Min(cm)	204.29	175.91	207.07	187.5	206.72
				EH		
TC(Zheng 58)	Mean(cm)	94.85	68.99	93.93	85.17	85.97
	Max(cm)	125.00	107.52	117.57	112.75	110.61
	Min(cm)	72.38	50.03	64.23	63.96	66.89
	*r*	0.82**	0.78**	0.85**	0.82**	0.84**
TC(Chang 7–2)	Mean(cm)	121.77**	94.36**	119.44**	115.19**	110.42**
	Max(cm)	151.67	118.71	141.17	163.38	132.83
	Min(cm)	85.95	79.84	89.17	90.63	86.33
	*r*	0.71**	0.71**	0.80**	0.71**	0.83**

^**^ *P* ≤ 0.01

^a^Comparison between average performance of TC (Zheng 58) and TC (Chang 7–2) for PH and EH

*r*: Correlation between PH and EH in each population

Variance analysis of Z_1_ and Z_2_ revealed that *V*_*A*_ and *V*_*D*_ for PH and EH were significant (*P* < 0.01) (Table 3). We calculated the average degree of dominance (D*) for each trait. The results showed that the D* was 0.83 for PH and 0.71 for EH. In addition, broad sense heritability (*h*_*B*_^*2*^) was high for PH and EH in both Z_1_ and Z_2_ (0.77 ~ 0.94). Remarkably, the *h*_*B*_^*2*^ was higher in Z_1_ than that in Z_2_ for PH and EH.

**Table 3:** *V*_*A*_, *V*_*D*_, broad sense heritability (*h*_*B*_^*2*^) and average degree of dominance (D*) for Z_1_ and Z_2_ across five environments

Linear transformations	Parameter	Trait	
		PH	EH
Z_1_	*V*_*A*_^a^	1543.45^**^	762.28^**^
	*h*_*B*_^*2*^	0.94	0.94
	CI (*h*_*B*_^*2*^)^c^	(0.93, 0.95)	(0.92, 0.95)
Z_2_	*V*_*D*_^b^	2134.61^**^	769.06^**^
	*h*_*B*_^*2*^	0.86	0.77
	CI (*h*_*B*_^*2*^)	(0.83,0.89)	(0.72,0.81)
	D*	0.83	0.71

^*^ *P* ≤ 0.05; ^**^ *P* ≤ 0.01

^a^additive variance; ^b^dominance variance; ^c^95% confidence interval

### Mapping environmentally stable QTLs for Z_1_ and Z_2_

**Fig. 2:**
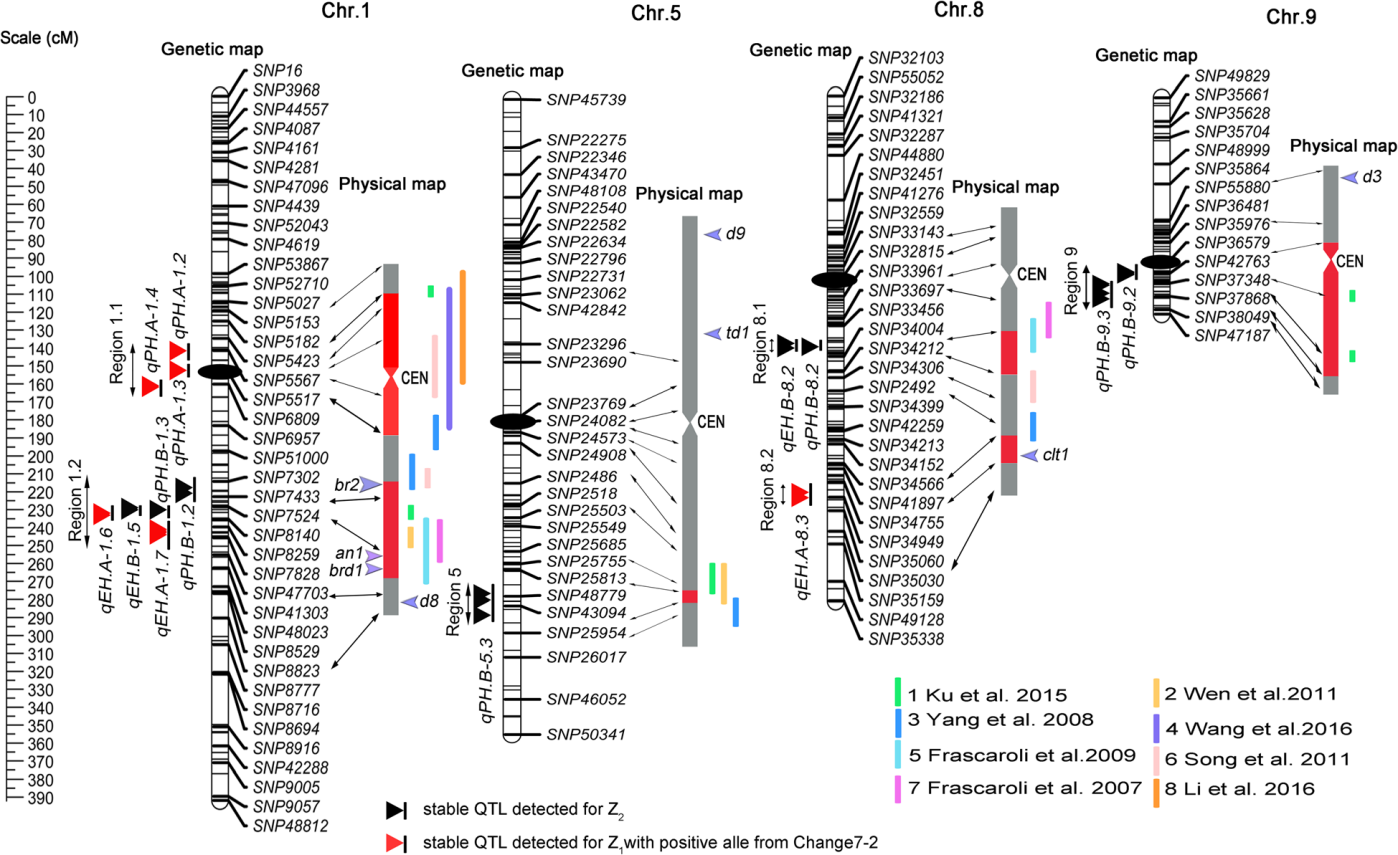
Genetic locations of the 14 environmentally stable QTLs for PH and EH. The centiMorgan (cM) scale is shown on the left. *Black* ellipses indicate the approximate positions of the centromeres. Vertical bars in *black* represent the confidence interval of each QTL. A *black vertical bar* with *black triangle* represents heterotic-related QTLs detected for Z_2_; a *black vertical bar* with a *red triangle* represents additive QTLs with positive alleles from parent Chang 7–2. Double-headed *arrows* represent the genomic regions characterized by QTL or QTL clusters. *Red shadows* on the physical map indicate the corresponding positions of each QTL. The verticals in different colors alongside the physical map indicate known heterotic-related QTLs from different studies (1 Ku et al. [43]; 2 Wen et al. [48]; 3 Yang et al. [45]; 4 Wang et al. [18]; 5 Frascaroli et al. [46]; 6 Song et al. [22]; 7 Frascaroli et al. [23]; 8 Li et al. [16]). The known positions of *br2*, *an1*, *brd1*, *d8*,*d9*, *td1*, *clt1*and *d3* are presented in *blue arrows*

A total of 111 QTL were detected for PH and EH in the maize hybrid Zhengdan 958 (Additional file 2). In the present study, a QTL identified within two or more environments as well as in the combined analysis was regarded as “environmentally stable QTL”. As a result, 14 environmentally stable QTLs were detected, which distributed on chromosomes 1, 5, 8 and 9 (Fig. 2 and Table 4).

**Table 4:** Genomic regions harboring environmentally stable QTL for plant height (PH) and ear height (EH) for Z_1_ and Z_2_

Genomic regions^a^	Interval (cM)	Associated traits^b^	Included QTL	Z_1_^c^			Z_2_			Gene cation^d^	Detected environment^e^	References^f^
				LOD	*a*_*i*_ *	*R* ^*2*^ (%)	LOD	*d*_*i*_ *	*R* ^*2*^ (%)			
Region 1.1	137.7-167.4	PH (+)	*qPH.A-1.2*	4.6	−3.2	8.6				PD	E4,E5,C	1,4
		PH (+)	*qPH.A-1.3*	6.3	−3.8	11.5				PD	E1,E3,E4,E5,C	4,6
		PH (+)	*qPH.A-1.4*	4.6	−3.2	8.5				A	E4,E5,C	3,4,6,8
**Region 1.2**	212.6-253.8	PH	*qPH.B-1.2*				3.7	2.6	5.4	OD	E1,E4,C	3,6
		PH	*qPH.B-1.3*				3.6	2.5	5.2	OD	E1,E4,C	1
		EH	*qEH.B-1.5*				5	2	8.4	OD	E4,E5,C	2
		EH (+)	*qEH.A-1.6*	4	−2	7.6				D	E1,E2,E3,E4,C	2,5,7
		EH (+)	*qEH.A-1.7*	4.5	−2.1	8.5				D	E1,E2,E3,C	5,7
Region 5	271.6-292.5	PH	*qPH.B-5.3*				4.7	3	7.2	OD	E2,E4,C	1,2,3
**Region 8.1**	136.2-143.3	PH	*qPH.B-8.2*				6.6	3.6	9.9	OD	E1,E4,E5,C	5,6,7
		EH	*qEH.B-8.2*				5.7	2.2	9.5	OD	E3,E4,C	5,6,7
Region 8.2	215.7-228.3	EH (+)	*qEH.A-8.3*	4.9	−2.2	9.2				A	E2,E3,E4,E5,C	3
Region 9	96.5-122.1	PH	*qPH.B-9.2*				6.9	4.9	10.2	OD	E2,E3,E5,C	1
		PH	*qPH.B-9.3*				6.3	4.4	9.4	OD	E2,E3,E4,C	1

^a^The genomic regions shown in bold are the ones with pleiotropic effect

^b^Traits are plant height (PH) and ear height (EH). The plus (“+”) signs within the brackets indicate Chang 7–2 contributed increasing alleles

^c^QTL information for the combined analysis

^d^Degree of dominance: A, additive (|*d*_*i*_ */*a*_*i*_ *| ≤ 0.20); PD, partial dominance (0.20 < | *d*_*i*_ */*a*_*i*_ *| < 0.80); D, dominance (0.80 ≤ |*d*_*i*_ */*a*_*i*_ *| < 1.20); and OD, overdominance (|*d*_*i*_ */*a*_*i*_ *| ≥ 1.20)

^e^C Indicates the combined QTL analysis based on the BLUP values across five environments

^f^Heterosis-associated QTLs reported in previous studies: 1 Ku et al. [43]; 2 Wen et al. [48]; 3 Yang et al. [45]; 4 Wang et al. [18]; 5 Frascaroli et al. [46]; 6 Song et al [22]; 7 Frascaroli et al. [23]; 8 Li et al. [16]

Fifty-six QTLs associated with PH were detected. Nine environmentally stable QTLs for PH were identified on chromosomes 1, 5, 8 and 9, which were designated *qPH.A-1.2*, *qPH.A-1.3*, *qPH.A-1.4*, *qPH.B-1.2*, *qPH.B-1.3*, *qPH.B-5.3*, *qPH.B-8.2*, *qPH.B-9.2* and *qPH.B-9.3,* respectively. Parental line Chang 7–2 contributed PD effect for the increased PH of *qPH.A-1.2* and *qPH.A-1.3*, as well as A effect for the increased PH of *qPH.A-1.4*, for the combined analysis. The rest six QTLs showed an OD effect for Z_2_, which explained from 5.2 to 10.2% of variation for the combined analysis.

Fifty-five QTLs were found to be associated significantly with EH, and five environmentally stable QTLs were detected on chromosomes 1 and 8 (*qEH.A-1.6*, *qEH.A-1.7*, *qEH.A-8.3*, *qEH.B-1.5* and *qEH.B-8.2*). Parental line Chang 7–2 contributed D effect for the increased EH of *qEH.A-1.6* and *qEH.A-1.7*, which explained 7.6 and 8.5% of variation for the combined analysis, respectively. In addition, Chang 7–2 contributed increased effects for *qEH.A-8.3* with additive effect (A) and explained 9.2% of variation for the combined analysis. Remarkably, *qEH.B-1.5* and *qEH.B-8.2* exhibited an OD effect for Z_2_ and explained 8.4 and 9.5% of variation for the combined analysis, respectively.

Interestingly, the 14 environmentally stable QTLs were anchored on six genomic regions (Fig. 2 and Table 4). Region 1.1 contained three tightly linked QTLs for PH (*qPH.A-1.2*, *qPH.A-1.3* and *qPH.A-1.4*), and each of them was detected in Z_1_. Region 1.2 covered two overdominant QTLs for PH (*qPH.B-1.2* and *qPH.B-1.3*), one overdominant QTL for EH (*qEH.B-1.5*) and two dominant QTLs for EH (*qEH.A-1.6* and *qEH.A-1.7*). Region 8.1 harbored two overlapped dominant QTLs, with one for PH (*qPH.B-8.2*) and the other one for EH (*qEH.B-8.2*). Region 9 contained two tightly linked dominant QTLs for PH (*qPH.B-9.2* and *qPH.B-9.3*). In addition, region 5 and region 8.2 mapped a single QTL for PH (*qPH.B-5.3*) and EH (*qEH.A-8.3*), respectively.

### Validation of *qPH.A-1.3*

**Fig. 3:**
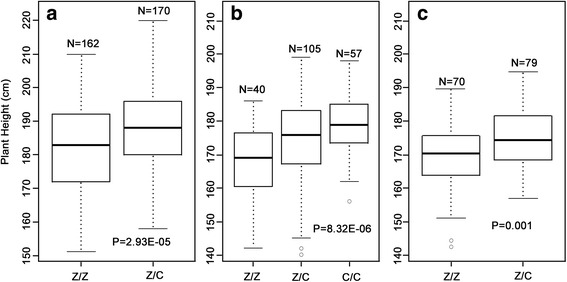
Validation of *qPH.A-1.3* for plant height (PH) in a: BC_2_F_1_ population, b: BC_2_F_2_ population, c: BC_3_F_1_ population. The three populations were genotyped by using the SSR markers MPH72 and MPH1149. The distributions and mean values for PH are shown as different genotypic classes: Z/Z homozygous for Zheng 58 haplotype, C/C homozygous for Chang 7–2 haplotype, or Z/C for heterozygous

*qPH.A-1.3* could be mapped in four of the five investigated environments and explained a large amount of phenotypic variance (Table 4), which revealed its potential for further study. Thus, we developed ten polymorphic SSR markers to validate the presence of *qPH.A-1.3*, and all of them were anchored on the *qPH.A-1.3* region based on the RIL population (Additional file 3 and Table 5). Two SSR markers (MPH72 and MPH1149) flanking *qPH.A-1.3* were used to determine the individual genotypes in BC_2_F_1,_ BC_3_F_1_ and BC_2_F_2_. The genotype which was identical to the Zheng 58 parent was designated Z/Z, the genotype which was identical to the Chang 7–2 parent was designated C/C, while the genotype which carried both Zheng 58 and Chang 7–2 parental alleles was designated Z/C. Of the plants in BC_2_F_1_ and BC_3_F_1_, the average PH of Z/C individuals were 6.1 and 5.2 centimeters higher than that of Z/Z (*t*-test, *P* = 2.93E-05 and *P* = 0.001, respectively). While in the BC_2_F_2_ population, PH differed significantly (*F*-test, *P* = 8.32E-06) between the three genotypic classes. The average PH of C/C plants was 11.7 centimeters higher than that of Z/Z (Fig. 3).

**Table 5:** Newly developed SSR markers for *qPH.A-1.3* region

Marker	Forward primer(5'-3')	Reverse primer(5'-3')	Annealing temperature (°C)
MPH72	CTGGGAAGGAAACCTAAACA	CGACTGAGGACACCTATAGACA	58
MPH96	GTTGCCTTGTTCTTGATTCAC	TAGCTGCCAGTGGTACTTTTC	60
MPH1056	TATCCGCTTTCTTCCCTTCT	ACCGCAACCATTCAACATAC	58
MPH1042	CCGCTTTCTTCCCTTCTCTT	CCGCAACCATTCAACATACA	58
MPH1061	CGCGTAAGTTGTGTGTTTTT	TCTTTTAGTTGAGGCCATTC	57
MPH1088	GCACGCAAGAGAGGAATAGA	AAGAGGGAGGATGAGGATTA	58
MPH16	AGGAGCTAGGGATTGAATATG	GAATTTGACCCGAATTTCC	58
MPH1132	CCTGTCAGAGACAGTTCTC	GAGAGAAGAAAAGGGGTACG	59
MPH1149	GAACATACCAGTATGGAAGGA	GACCAAATTGGGACTTAACC	58
MPH5324	TCCAAGTGACAGAATAAACTTTC	ATCACAAGGGTCATCTTCCG	58

To estimate the degree of dominance, we also compared PH of the three genotypic classes at *qPH.A-1.3* in BC_2_F_2_ population. The additive effect was 5.85 centimeters. The degree of dominance obtained was 0.11, which indicated additive gene action (Fig. 3 and Table 6). Collectively, the statistically significant difference of PH in BC_2_F_1,_ BC_3_F_1_ and BC_2_F_2_ indicated an effect of genotype in the *qPH.A-1.3* region on PH phenotype.

**Table 6:** Gene action of *qPH.A-1.3* for plant height (PH) in BC_2_F_2_ population

Trait	Genotypic classes			Number
	Z/Z	Z/C	C/C	
PH	167.9	174.4	179.6	202

Genotypic classes obtained by the two markers (MPH72 and MPH1149) flanking *qPH.A-1.3* region. Z/Z indicates homozygous for Zheng58, Z/C indicates heterozygous, and C/C indicates homozygous for Chang7-2

## Discussion

### Genetic basis of heterosis underlying PH and EH

Plant height and ear height are decisive factors to plant density and lodging resistance [20, 40]. In this study, we studied the genetic basis underlying PH and EH with a RIL based design III. In total, 111 QTLs were identified (Additional file 2), which indicates the highly polygenic inheritance pattern underlying PH and EH. Interestingly, all the eight environmentally stable QTLs for Z_2_ were characterized by OD effects, which is consistent with the results in Song et al. [22], suggesting that overdominant QTLs are important contributors to PH and EH. Nevertheless, QTL showed high overdominance effect may be the result of linked dominant QTLs in repulsion. For example, Graham et al. dissected a major overdominant QTL on chromosome 5 into two dominant QTLs with repulsion-phase linkage [41]. More recently, Li et al. reported that two separate loci with a repulsion linkage could appear as a single locus with an overdominance mode of inheritance [42].

Four of the six environmentally stable QTLs from Z_1_ exhibited PD or D effects. However, the dominance degree was incompatible in different environments for a given QTL detected for Z_1_. For example, *qPH.A-1.3* showed additive effect (A) in E1, while showed PD effect in other environments (Additional file 2). In addition, this QTL exhibited additive effect (A) in a BC_2_F_2_ population (Fig. 3 and Table 6). Therefore, we assume that the expression of the degree of dominance might be affected by the environment and/or the genetic background, which will be an interestingly area for further investigation.

### Comparison of QTL regions with previous studies

Due to the high heritability and the ease of its measurement, PH and EH have been analyzed in different studies, and common QTLs were reported between individual populations, which provided valuable information for future studies including their positional cloning or marker-assisted selection [16, 43, 44]. The present study identified six genomic regions on four chromosomes, which harbor 14 environmentally stable QTLs for PH and EH (Fig. 2 and Table 4). Comparison analysis revealed that the detected genomic regions overlapped with previous reported QTLs in terms of PH and EH, and the percentage of overlapping for each QTL varied from 1.4 - 100% [16, 18, 22, 23, 45–48] (Fig. 2 and Additional file 4). For example, region 1.2 appeared to be involved in PH and EH with D or OD effects, and it was reported to be a dominant QTL for PH in studies of Frascaroli et al. [23, 46] as well as an additive QTL for EH in study of Yang et al. [45]. The heterosis associated region on chromosome 8 (region 8.1) that showed high individual *R* ^*2*^ for both PH and EH was detected as QTL for PH in Frascaroli et al. and Song et al. [22, 23, 46]. Notably, considering the low percentage of overlapping for some QTLs with previous studies, the reliability of overlapped QTLs across different studies deserves for further evaluation.

Region 1.1 contained three tightly linked QTLs for PH, whereas no QTLs were detected for EH. QTLs controlling both PH and EH in this region were reported in several studies [18, 22, 45]. Moreover, a QTL for internode length above the uppermost ear was detected by using four RIL populations [47]. Taken together, the lack of QTL for EH in the present study could be ascribed to the following reasons: 1) different allelic variations of the same gene lead to different phenotypes; 2) different genes for PH and /or EH existed in this QTL region.

### The relationship between PH and EH

Our results showed that positive correlation coefficients between TCs were observed for PH and EH (Table 2). PH is composed of internode number and length both up and below the ear, and EH is composed of internode number and length below the ear. Theoretically, some QTLs might exhibit pleiotropic effects to the two traits. As expected, two overlapped QTLs for PH and EH were identified (Fig. 2 and Table 4). For example, region 1.2 contains one overdominant QTL for PH (*qPH.B-1.3*), one overdominant QTL for EH (*qEH.B-1.5*) and one dominant QTL for EH (*qEH.A-1.6*); region 8.1 contains two overdominant QTLs for PH and EH (*qPH.B-8.2* and *qEH.B-8.2*). However, the possibility of tightly linked QTLs, each controlling PH or EH, could not be ignored.

Notably, some trait-specific QTLs are identified, that is some regions harbor QTLs only for PH or EH, such as *qPH.B-5.3* in region for PH and *qEH.A-8.3* in region 8.2 for EH. Similar phenomenon was reported in previous studies [47–49]. For example, Ku et al. reported common and position-specific QTLs affecting internode length at different positions above the uppermost ear [47]. Li et al. demonstrated that the number of leaves above and below the primary ear were under relatively independent genetic control [49]. In conclusion, these results suggested that the underlying genetic basis for PH and EH is partially different, which will be an interesting area for further study.

### Candidate genes in QTL Regions for PH and/or EH

To date, over 40 dwarf or semi-dwarf genes have been identified in maize (http://www.maizegdb.org/data_center/phenotype?id=11041/). To identify the candidate genes located within QTLs for PH and/or EH, we firstly analyzed the relationship of maize dwarf or semi-dwarf genes with the detected QTLs for PH and/or EH in this study. Interestingly, four genes were found to locate in QTL regions, including *an1*, *brd1* and *br2* in genomic region 1.2 and *ctl1* in region 8.2 (Fig 2 and Additional file 5). AN1 controls a step before ent-kaurene formation, which responds to gibberellins [50]; BRD1 is a homolog encoding brC-6 oxidase, an enzyme that controls the last steps of brassinosteroid biosynthesis [51]. BR2 is an ABC (ATP-binding cassette) transporter belongs to the MDR (multi-drug resistant) class of P-glycoprotein and functions in polar auxin transport as an efflux carrier [52, 53]. CLT1 was reported to code a BTB domain-containing protein that comprises 745 amino acids by bioinformatics analysis [54].

Gibberellin (GA) and brassinosteroid (BR) pathways play key roles in the control of plant height [19, 20]. Thus, we also surveyed the candidate genes responsible for the two pathways in QTL Regions for PH and/or EH. As shown in additional file 5, two GA biosynthesis genes (GRMZM2G117940 and GRMZM2G164090) were found in region 1.1 and region 8.1, respectively. In addition, two GA and one BR pathway genes, including GRMZM2G059308 for GA biosynthesis, GRMZM2G114680 for GA signaling and GRMZM2G424075 for brassinosteroid biosynthesis located in region 9. Notably, *br2* and GRMZM2G164090 were co-localized with PH and EH QTLs (Additional file 5). A rare SNP mutation in *br2* could affect PH and EH by reducing average internode length and internode number [19]. However,the function of gene GRMZM2G164090 on PH and EH has not been characterized. Collectively, those genes are possible candidates for the detected PH and EH QTLs, and detailed studies would be necessary to evaluate their relationship with the QTLs identified in the present study.

### Potential utilization of *qPH.A-1.3* in maize breeding

Although many dwarf genes for PH have been cloned, the unacceptable impact on yield production restrained their further utilizations in maize breeding [4]. Therefore, dwarf genes/QTLs with no or very little negative impact on grain yield can be considered as good genetic resources for breeding [19, 20]. This mapping study identified a QTL-*qPH.A-1.3* for PH, which stably showed up in four environments as well as in the combined analysis. Further investigation of three backcross populations (BC_2_F_1_, BC_2_F_2_ and BC_3_F_1_) validated the real existence of this additive QTL and revealed that *qPH.A-1.3* modified about 10 centimeters of PH. Furthermore, when comparing with the results of QTLs for ear-weight-related traits which adopted the same genetic materials, including ear row number, ear diameter, number of seeds per row, ear length, one hundred seed weight, ear seed number, ear seed weight and ear weight, overlaps were found in region 1.2, 8.1 and 9. However, no QTL for the ear-weight-related traits was detected in the QTL region of *qPH.A-1.3* (Additional file 6) [55]. Collectively, we proposed that *qPH.A-1.3* may be useful in maize breeding without altering grain yield by using marker-assisted selection for two purposes: 1) For energy production and chemical feedstocks, the utilization of Chang 7–2 allele could increase plant height and biomass; 2) For lodging resistance, the Zheng 58 allele could be used to decrease plant height. However, the relationship between *qPH.A-1.3* and ear-weight-related traits should be precisely evaluated by advanced segregating populations, such as near-isogenic lines, which is currently underway.

## Conclusions

Of 14 environmentally stable QTLs identified by design III populations, the eight heterosis associated environmentally stable QTLs exhibited OD effects, suggesting that overdominant QTLs were the most important contributors to heterosis for PH and EH. In addition, a major QTL *qPH.A-1.3* was confirmed to modify about 10 centimeters of PH, which may be a desired target for genetic improvement of plant height.

## Additional files

Additional file 1: Detailed information of the five environments in which the materials were evaluated. (DOC 630 kb)

Additional file 2: Genotypic data of the Zhengdan 958 RIL population that used in QTL analysis. (XLS 2128 kb)

Additional file 3: QTLs detected for plant height (PH) and ear height (EH) in Z_1_ and Z_2_ in five environments (XLS 45 kb)

Additional file 4: The genetic linkage map before and after encryption of *qPH.A-1.3* region. (DOC 663 kb)

Additional file 5: Comparison analysis of detected QTLs for plant height (PH) and/or ear height (EH) with previous studies. (XLS 34 kb)

Additional file 6: Candidate genes in QTL regions. (XLS 30 kb)

Additional file 7: Comparison of QTLs for PH (plant height) and EH (ear height) with QTLs for ear-weight-related traits in our previous study. (DOC 3075 kb)

## Abbreviations

A: Additive effect
*D**: Average degree of dominance
D: Dominance effect
EH: Ear height
MPH: Mid-parent heterosis
OD: Overdominance effect
PD: Partial dominance effect
PH: Plant height
QTL: Quantitative trait locus
RIL: Recombinant inbred line
TC: Testcross
