# Supplementary material for: Quantitative trait locus analysis of heterosis for plant height and ear height in an elite maize hybrid zhengdan 958 by design III
Source: BMC Genet. 2017 Apr 17;18:36. doi: 10.1186/s12863-017-0503-9 (PMC5392948; doi:10.1186/s12863-017-0503-9)
Supplement: Supplementary file 1 — Detailed information of the five environments in which the materials were evaluated. (DOC 630 kb) [file 12863_2017_503_MOESM1_ESM.doc]

Table. Environments in which the TCs, RILs and the basic generations (the parental line Zheng 58, Chang 7-2 and the hybrid Zhengdan 958) were evaluated.

| Environment | Location | Growing season | Latitude, longitude |
| --- | --- | --- | --- |
|
| E1 | Gongzhuling, Jilin | 2012 | 124°81' E, 43°51' N |
| E2 | Jinghai, Tianjin | 2012 | 116°92' E, 39°03' N |
| E3 | Wulumuqi, Xinjiang | 2012 | 87°36' E, 43°46' N |
| E4 | Wulumuqi, Xinjiang | 2013 | 87°36' E, 43°46' N |
| E5 | Gongzhuling, Jilin | 2013 | 124°81' E, 43°51' N |


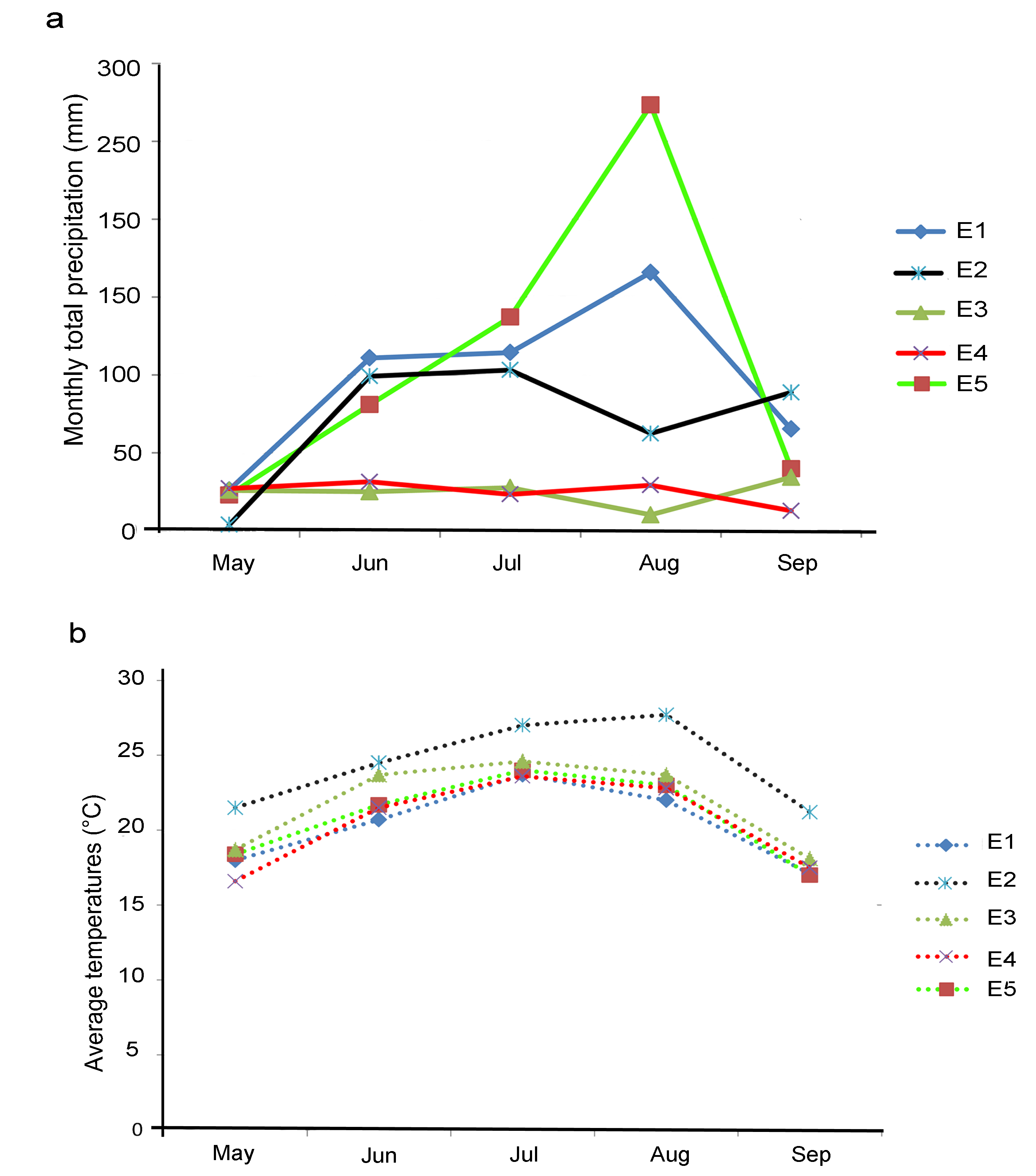


Fig. (a) Monthly total precipitation across five environments. (b): Monthly average temperatures across five environments.
