# Supplementary material for: Quantitative trait locus analysis of heterosis for plant height and ear height in an elite maize hybrid zhengdan 958 by design III
Source: BMC Genet. 2017 Apr 17;18:36. doi: 10.1186/s12863-017-0503-9 (PMC5392948; doi:10.1186/s12863-017-0503-9)
Supplement: Supplementary file 4 — The genetic linkage map before and after encryption of qPH.A-1.3 region. (DOC 663 kb) [file 12863_2017_503_MOESM4_ESM.doc]

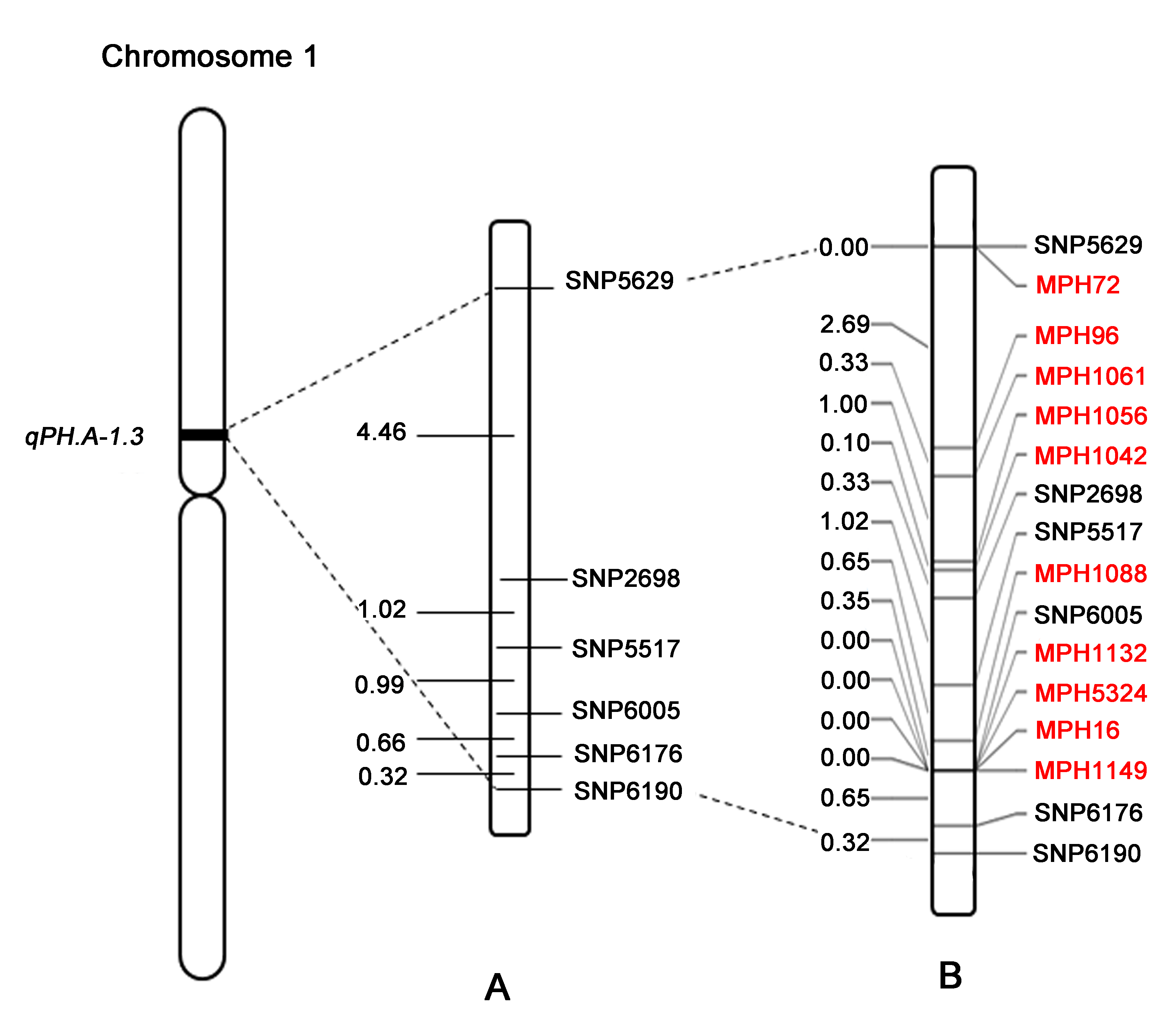


The genetic linkage map before and after encryption of *qPH.A-1.3* region. A: SNP orders for primary mapping; B: Markers in red indicate newly developed SSR markers.
