# Supplementary material for: Quantitative trait locus analysis of heterosis for plant height and ear height in an elite maize hybrid zhengdan 958 by design III
Source: BMC Genet. 2017 Apr 17;18:36. doi: 10.1186/s12863-017-0503-9 (PMC5392948; doi:10.1186/s12863-017-0503-9)
Supplement: Supplementary file 7 — Comparison of QTLs for PH (plant height) and EH (ear height) with QTLs for ear-weight-related traits in our previous study. (DOC 3075 kb) [file 12863_2017_503_MOESM7_ESM.doc]

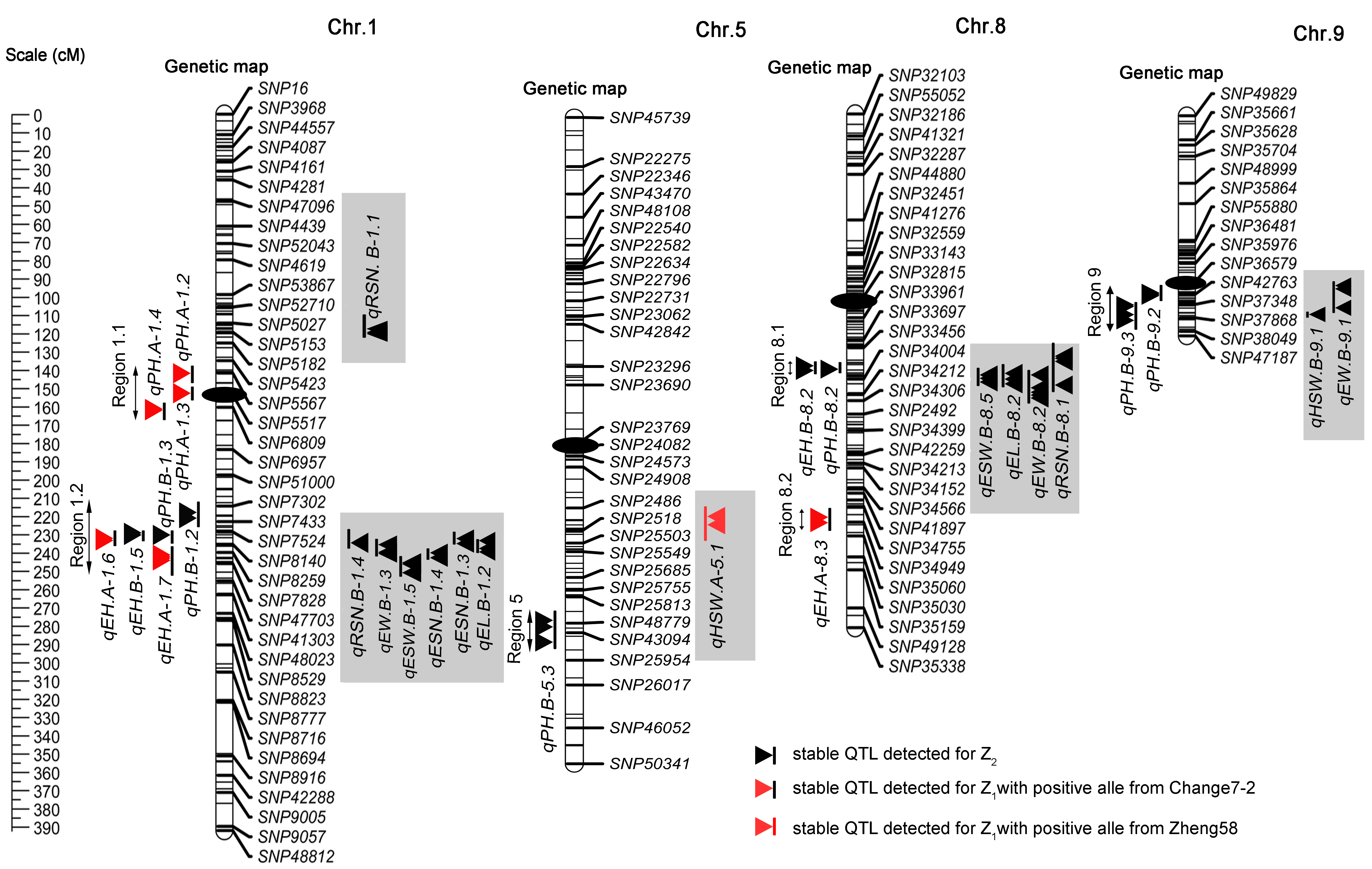


Fig. Comparison of QTLs for PH (plant height) and EH (ear height) with QTLs for ear-weight-related traits in our previous study, which adopted the same genetic materials (Li et al. 2017). QTLs for PH and EH are placed on the left side of each genetic map, while QTLs for ear-weight-related traits (in shadow) are placed on the right side of each genetic map. Ear-weight-related traits are ear row number (ERN), ear diameter (ED), number of seeds per row (RSN), ear length (EL), one hundred seed weight (HSW), ear seed number (ESN), ear seed weight (ESW) and ear weight (EW).
